# Treatment outcomes and radiotherapy deintensification strategies in human papillomavirus-associated tonsil cancer

**DOI:** 10.1186/s13014-022-02177-1

**Published:** 2022-12-20

**Authors:** Joongyo Lee, Kangpyo Kim, Kyung Hwan Kim, Ki Chang Keum, Hye Ryun Kim, Min Hee Hong, Eun Chang Choi, Se-Heon Kim, Yoon Woo Koh, Chang Geol Lee

**Affiliations:** 1 Department of Radiation Oncology, Yonsei Cancer Center, Heavy Ion Therapy Research Institute, Yonsei University College of Medicine, 50-1 Yonsei-Ro, Seodaemun-Gu, Seoul, 03722 Republic of Korea; 2 Division of Medical Oncology, Department of Internal Medicine, Yonsei Cancer Center, Yonsei University College of Medicine, 50-1 Yonsei-Ro, Seodaemun-Gu, Seoul, 03722 Republic of Korea; 3 Department of Otorhinolaryngology, Yonsei University College of Medicine, 50-1 Yonsei-Ro, Seodaemun-Gu, Seoul, 03722 Republic of Korea

**Keywords:** Tonsil cancer, Radiotherapy, Human papillomavirus

## Abstract

**Background:**

Human papillomavirus (HPV)-positive tonsil cancer has a better prognosis than HPV-negative tonsil cancer. Deintensification strategies to reduce or avoid radiotherapy (RT) for patients with HPV-associated tonsil cancer have been suggested. This study investigated the treatment outcomes of patients with HPV-associated tonsil cancer and suggested RT deintensification strategies.

**Methods:**

A cohort of 374 patients with HPV-associated tonsil cancer treated with primary surgery or RT between 2008 and 2020 was retrospectively evaluated. Survival and locoregional control rates after primary surgery or RT were analyzed, and propensity score matching was performed to adjust for clinical factors. Pearson's chi-square or Fisher's exact test was used to compare categorical variables, and Student's t-test was used to compare continuous variables. The Kaplan–Meier method and log-rank test were used to assess overall survival, progression-free survival, and locoregional failure (LRF).

**Results:**

No significant differences in survival or LRF were observed between the primary surgery and RT groups. Subgroup analysis was conducted for patients who underwent primary surgery. Advanced pathological N stage, negative contralateral nodes at diagnosis, abutting or positive surgical margins, and no adjuvant RT were independent risk factors for LRF. Advanced pathological T stage was an independent risk factor for LRF in patients who underwent primary surgery without subsequent adjuvant RT. None of the patients with pathological complete remission (CR) after induction chemotherapy died or experienced LRF.

**Conclusions:**

Our study revealed that the outcomes of primary surgery and primary RT in HPV-positive tonsil cancer were similar after adjusting for clinical factors. Primary RT might be considered instead of surgery in patients with advanced pathological T stage. In the case of pathological CR after induction chemotherapy, deintensification for adjuvant RT should be considered.

**Supplementary Information:**

The online version contains supplementary material available at 10.1186/s13014-022-02177-1.

## Introduction

Human papillomavirus (HPV)-positive oropharyngeal cancer accounts for 60–70% of all oropharyngeal cancers [1] and has a better prognosis than HPV-negative oropharyngeal cancer, with a risk of death nearly half that of HPV-negative oropharyngeal cancer [2]. Standard treatments, including radiotherapy (RT), have shown high local control rates; however, many treatment-related toxicities have been reported [3]. Several deintensification strategies have been suggested to reduce the treatment intensity.

RT is used as definitive or adjuvant therapy in patients with HPV-associated oropharyngeal cancer. Deintensification strategies for primary RT that reduce the prescription dose for concurrent chemoradiotherapy (CCRT) [4, 5], sequential RT to patients with a good response after induction chemotherapy [6], and administering RT alone without concurrent chemotherapy [7] produced similar results to standard treatment. Additionally, deintensification strategies for adjuvant RT, such as performing surgery alone without adjuvant RT in low-risk patients [8], reducing the prescription dose [9], or reducing the field of adjuvant RT [10], have been suggested. However, evidence and guidelines for RT deintensification strategies have not yet been established.

Among patients with the same type of oropharyngeal cancer, the pathophysiology differs according to tumor subsite. For example, HPV is more often positive in cancers of the tonsil than those at the base of the tongue [11]. Additionally, bilateral or contralateral cervical lymph node (LN) metastasis is more common in HPV-positive cancer at the base of the tongue than that in the tonsils [12]. However, most studies of deintensification strategies for HPV-associated oropharyngeal cancer do not take the subsite into account.

Herein, treatments for HPV-associated tonsil cancer are compared, and strategies for deintensification are presented. Outcomes according to treatment modality and deintensification strategies for RT were also investigated.

## Materials and methods

### Patient selection

We identified 395 patients with histologically proven HPV-associated tonsil cancer in clinical stages T1–4, N0–3 (American Joint Committee on Cancer, 8th Edition), who were treated with primary surgery or RT at our institution between 2008 and 2020. The exclusion criteria were as follows: (1) double primary cancers (n = 7); (2) incomplete RT regimen (n = 8); or (3) no records of RT (n = 6). Finally, 374 patients were included in our cohort.

This study was approved by the Institutional Review Board of our University (No. 4-2021-1332). The requirement for informed consent was waived because of the retrospective nature of the study. All procedures were conducted in accordance with the 2000 revision of the Declaration of Helsinki.

### Treatment modality

All patients underwent primary RT or surgery of curative intent, as decided by a multidisciplinary team. Primary RT was performed in patients with locally advanced cancer, older patients, or patients in whom surgical resection was difficult to perform; primary surgery was performed otherwise. Based on histological findings, adjuvant RT was administered after primary surgery if necessary. In some cases, chemotherapy was induced to reduce the extent of the disease, followed by a transoral robotic surgery (TORS).

Tonsillectomy was performed in patients who underwent surgery; TORS was performed according to the disease extent. Patients also underwent ipsilateral or bilateral neck dissection, depending on their disease status.

All patients who received RT underwent simulation computed tomography (CT) for RT planning. During simulation CT, the patients’ head and neck were immobilized with a thermoplastic mask in the supine position. Clinical target volume 1 (CTV1) was defined as gross lesions of the tonsils and LNs for definitive therapy and surgical beds of the tonsils and positive LNs for adjuvant therapy; CTV2, as the involved elective neck; and CTV3, as the uninvolved elective neck. The planning target volume was defined as the CTV plus 3-mm margins. The Pinnacle system (Philips Medical Systems, Cleveland, OH, USA) was used for three-dimensional conformal RT plans, and TomoTherapy (Accuray, Sunnyvale, CA, USA) or RayStation (RaySearch Laboratories, Stockholm, Sweden) was used for intensity-modulated RT plans. The equivalent dose at 2 Gy per fraction (EQD2) was calculated to compare different RT fractionation regimens using the following equation: EQD2 = D(α/β + d)/(α/β + 2), where D = total dose, d = dose per fraction, and α/β = 10 for tonsil cancer.

Chemotherapy was administered as induction therapy before primary local treatment or concurrently with RT; titanium silicate-1 and cisplatin were used for induction therapy, and cisplatin was used for CCRT.

### Follow-up

After treatment, patients were followed up clinically every 3 months for the first year, every 6 months for the next 4 years, and once a year thereafter. Chest CT and neck MRI were performed every 6 months for the first 5 years, and once a year thereafter, with additional imaging as indicated. Recurrence was determined by a comprehensive evaluation of MR images and clinical findings.

### Statistical analysis

Pearson's chi-square or Fisher's exact test was used for comparing categorical variables, and Student's t-test was used for comparing continuous variables. The Kaplan–Meier method and log-rank test were used to assess overall survival (OS), progression-free survival (PFS), and locoregional failure (LRF). Events were measured from the date of initial treatment. Deaths were not counted as LRF. Univariate and multivariate analyses were performed with Cox proportional hazards models. A multivariate analysis was conducted using backward stepwise selection.

Propensity score matching (PSM) analysis compared the treatment outcomes between the primary surgery and RT groups to adjust for clinical factors. Propensity scores were calculated using a multivariable logistic regression model with age, sex, tobacco use (≤ 10 pack-years vs. > 10 pack-years), T stage, and clinical N stage. Using nearest-neighbor matching with a caliper distance of 0.01 standard deviations of the logit of the propensity score, primary surgery and primary RT patients were matched 1:1 based on their scores. The standardized mean difference evaluated the balance of covariate distribution between the two groups.

Subgroup analyses were conducted for patients who underwent primary surgery to determine the difference in LRF according to adjuvant RT, risk factors for LRF in patients who underwent primary surgery without adjuvant RT, and prognostic differences according to pathological response after induction chemotherapy. Tumor response after induction chemotherapy was determined according to the Response Evaluation Criteria in Solid Tumors, version 1.1 [13].

Statistical significance was set at *p* < 0.05. IBM SPSS Statistics for Windows, version 26.0 (IBM Corp., Armonk, NY, USA) was used for statistical analysis.

## Results

### Baseline characteristics

The median age of the patients was 58 years [interquartile range (IQR, 52–63 years)]. Of the 374 patients, 323 (86.4%) were males. Further, 164 patients (43.9%) had a smoking history of > 10 pack-years. At the time of diagnosis, 75 patients (20.1%) had stage T3/4 cancer, and 47 (12.5%) had clinical stage N2/3 cancer.

Of the 374 patients, 84 (22.5%) received primary surgery alone, 224 (59.9%) received primary surgery plus adjuvant RT, and 66 (17.6%) received primary RT. Among 308 patients who underwent primary surgery, 194 (63.0%) underwent TORS. Of the remaining 114 patients who underwent primary surgery, 54 (17.5%) underwent a classical transoral approach, 38 (12.3%) underwent a mandibular incision approach, and 22 (7.1%) underwent a pharyngeal approach. Among 290 patients who received RT, 280 (96.6%) underwent intensity-modulated RT, and 235 (81.0%) underwent CCRT. The median total definitive doses of the CTV1, CTV2, and CTV3 were EQD2 67.1 Gy (IQR, 64.1–70.0 Gy), 53.1 Gy (IQR, 53.0–60.0 Gy), and 46.4 Gy (IQR, 41.5–50.8 Gy), respectively. The median total adjuvant doses of the CTV1, CTV2, and CTV3 were EQD2 60.0 Gy (IQR, 56.5–63.5 Gy), 53.1 Gy (IQR, 49.6–56.0 Gy), and 44.3 Gy (IQR, 44.0–47.2 Gy), respectively. Induction chemotherapy was administered to 123 patients (32.9%) in total, with 111 patients receiving chemotherapy before surgery. All patient characteristics are listed in Table 1.

**Table 1:** Baseline characteristics of total patients

Characteristics	Patients	
	N	%
Age (years, median [IQR])	58 (52–63)	
Sex		
Male	323	86.4
Female	51	13.6
Tobacco use		
≤ 10 pack-years	210	56.1
> 10 pack-years	164	43.9
Clinical T stage		
T1	87	23.2
T2	212	56.7
T3	44	11.8
T4	31	8.3
Clinical N stage		
N0	44	11.8
N1	283	75.7
N2	45	12.0
N3	2	0.5
Primary surgery	308	82.4
Mandibulotomy approach	38	12.3
Pharyngotomy approach	22	7.1
Classic transoral approach	54	17.5
TORS	194	63.0
Use of RT	290	77.5
Aim of RT		
Primary treatment	66	22.8
Adjuvant treatment	224	77.2
CCRT	235	81.0
Induction chemotherapy	123	32.9

*IQR* inter-quartile range, *TORS* transoral robotic surgery, *RT* radiotherapy, *CCRT* concurrent chemoradiotherapy

### Comparison between primary surgery and RT

The median follow-up duration was 47.0 (IQR, 25.7–75.2) months. The 2-year OS, PFS, and cumulative LRF rates for all patients were 94.2%, 82.2%, and 10.5%, respectively.

The primary surgery group consisted of patients with less advanced T and N stages and more heavy smokers than those in the primary RT group. After adjusting for propensity scores, the patient and tumor characteristics were well-balanced (Additional file 1: Table S1).

**Fig. 1:**
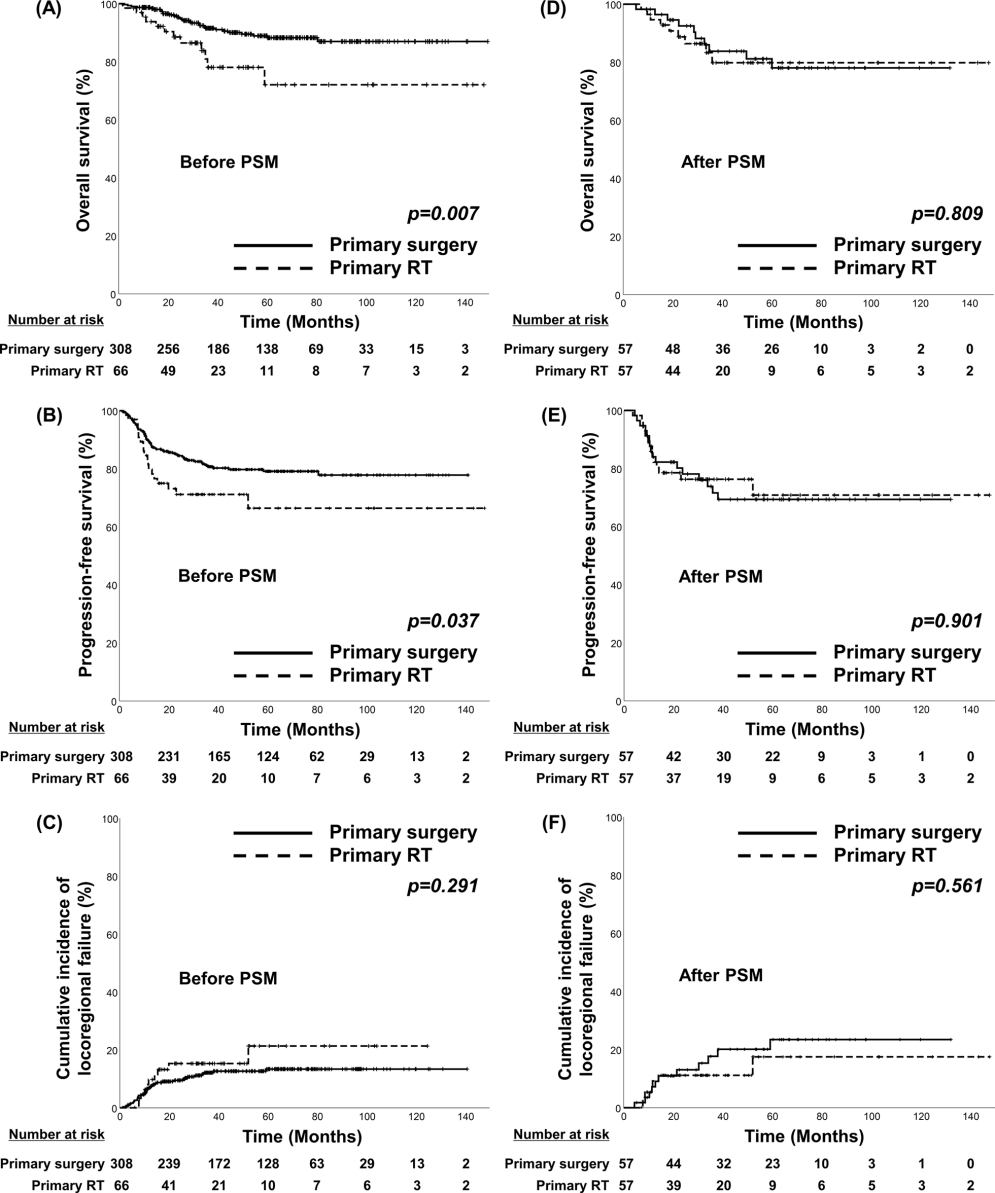
Kaplan–Meier curves for overall survival (OS), progression-free survival (PFS), and locoregional failure (LRF) according to the primary treatment before and after propensity score matching (PSM). (**A**) OS, (**B**) PFS, and (**C**) cumulative incidence of LRF for all patients. (**D**) OS, (**E**) PFS, and (**F**) cumulative incidence of LRF for matched patients. PSM, propensity score matching; RT, radiotherapy

Before PSM, OS and PFS were poorer in the primary RT group than in the primary surgery group (95.4% vs. 88.5%, *p* = 0.007 and 84.5% vs. 71.1%, *p* = 0.037, respectively; Fig. 1A, B). No statistical difference in LRF was observed between the groups (9.5% vs. 15.3%, p = 0.291) (Fig. 1C). Advanced clinical N stage [hazard ratio (HR, 3.25, *p* = 0.001)] for OS; male sex (HR, 0.36, *p* = 0.048), advanced T stage (HR, 1.81, p = 0.022), advanced clinical N stage (HR, 2.79, *p* < 0.001), and locoregional RT (HR, 0.57, *p* = 0.049) for PFS; smoking < 10 pack-years (HR, 0.49, *p* = 0.025); and advanced T stage (HR, 2.13, *p* = 0.027), advanced clinical N stage (HR, 3.70, *p* = 0.001), and locoregional RT (HR, 0.26, *p* < 0.001) for LRF were identified as poor prognostic factors through the multivariate analysis performed before PSM (Table 2).

**Table 2:** Prognostic factors for overall survival, progression-free survival, and locoregional failure before and after propensity score-matching

	Overall survival				Progression-free survival				Locoregional failure			
	Univariate analysis		Multivariate analysis		Univariate analysis		Multivariate analysis		Univariate analysis		Multivariate analysis	
	HR (95% CI)	*p* value	HR (95% CI)	*p* value	HR (95% CI)	*p* value	HR (95% CI)	*p* value	HR (95% CI)	*p* value	HR (95% CI)	*p* value
*Before PSM*												
Age (< 58 yrs vs. ≥ 58 yrs)	1.63 (0.86–3.08)	0.133			1.14 (0.73–1.78)	0.576			1.01 (0.56–1.79)	0.987		
Sex (Male vs. Female)	0.46 (0.14–1.50)	0.198			0.30 (0.11–0.83)	0.02	0.36 (0.13–0.99)	0.048	0.41 (0.13–1.31)	0.132	0.35 (0.11–1.18)	0.090
Tobacco use (≤ 10 PY vs. > 10 PY)	0.80 (0.42–1.51)	0.487			1.16 (0.74–1.81)	0.523			0.70 (0.38–1.28)	0.248	0.49 (0.27–0.91)	0.025
T stage (T1-2 vs. T3-4)	2.78 (1.48–5.20)	0.001	1.89 (0.95–3.77)	0.070	2.42 (1.52–3.87)	< 0.001	1.81 (1.09–3.00)	0.022	2.59 (1.42–4.72)	0.002	2.13 (1.09–4.15)	0.027
Clinical N stage (N0-1 vs. N2-3)	4.20 (2.20–8.02)	< 0.001	3.25 (1.59–6.61)	0.001	3.18 (1.93–5.26)	< 0.001	2.79 (1.58–4.91)	< 0.001	3.19 (1.68–6.08)	< 0.001	3.70 (1.70–8.05)	0.001
Primary site surgery (No vs. Yes)	0.41 (0.21–0.80)	0.009			0.58 (0.34–0.97)	0.038			0.69 (0.34–1.40)	0.303		
Locoregional radiotherapy (No vs. Yes)	1.60 (0.62–4.07)	0.329			0.82 (0.48–1.39)	0.466	0.57 (0.32–1.00)	0.049	0.45 (0.24–0.83)	0.010	0.26 (0.13–0.51)	< 0.001
Induction chemotherapy (No vs. Yes)	1.20 (0.61–2.36)	0.599			1.17 (0.73–1.89)	0.506			1.39 (0.76–2.55)	0.279		
*After PSM*												
Age (< 58 yrs vs. ≥ 58 yrs)	1.35 (0.53–3.44)	0.530			0.83 (0.40–1.73)	0.625			0.78 (0.31–1.98)	0.605		
Sex (Male vs. Female)	0.59 (0.14–2.54)	0.476			0.57 (0.17–1.89)	0.359			1.04 (0.30–3.58)	0.957		
Tobacco use (≤ 10 PY vs. > 10 PY)	1.29 (0.51–3.28)	0.593			1.29 (0.61–2.70)	0.507			0.59 (0.20–1.80)	0.356	0.37 (0.12–1.16)	0.089
T stage (T1-2 vs. T3-4)	3.00 (1.21–7.46)	0.018			3.18 (1.54–6.58)	0.002	2.09 (0.95–4.59)	0.065	4.83 (1.81–12.89)	0.002	3.76 (1.25–11.35)	0.019
Clinical N stage (N0-1 vs. N2-3)	5.21 (2.04–13.27)	0.001	5.21 (2.04–13.27)	0.001	4.60 (2.22–9.53)	< 0.001	3.47 (1.59–7.59)	0.002	4.29 (1.68–10.92)	0.002	2.51 (0.88–7.21)	0.087
Primary site surgery (No vs. Yes)	0.89 (0.36–2.22)	0.809			1.03 (0.50–2.12)	0.929			1.34 (0.51–3.47)	0.553		
Locoregional radiotherapy (No vs. Yes)	23.10 (0.02–2.82 × 10^4^)	0.387			1.28 (0.31–5.40)	0.733			0.72 (0.17–3.12)	0.658		
Induction chemotherapy (No vs. Yes)	1.99 (0.78–5.06)	0.150			1.36 (0.62–2.97)	0.446			1.29 (0.46–3.64)	0.628		

The foreparts of the parentheses were set as the reference groups in the multivariable analysis

*HR* hazard ratio, *CI* confidence interval, *PSM* propensity score-matching, *PY* pack-years

Following PSM, no statistical differences were observed in the 2-year OS, PFS, or LRF rates between the groups (92.5% vs. 88.7%, *p* = 0.809; 78.1% vs. 76.3%, *p* = 0.901; and 13.0% vs. 11.2%, *p* = 0.561, respectively) (Fig. 1D–F). A multivariate analysis performed after PSM identified the following poor prognostic factors: advanced clinical N stage for OS (HR, 2.79; *p* < 0.001) and PFS (HR, 2.79; *p* < 0.001) and advanced T stage (HR, 3.76; *p* = 0.019) for LRF (Table 2).

### Subgroup analysis

**Fig. 2:**
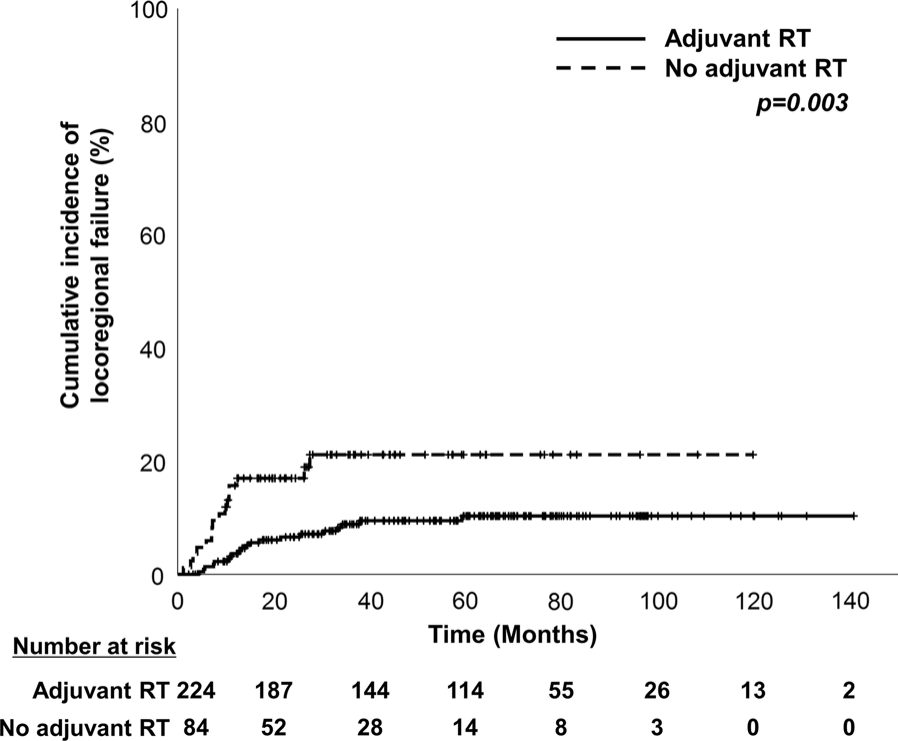
Cumulative incidence of locoregional failure according to adjuvant radiotherapy following primary surgery

Of the 308 patients who underwent primary surgery, 224 (72.7%) received adjuvant RT. Adjuvant RT was associated with a lower LRF; the 2-year LRF rates were 6.5% and 16.5% in the adjuvant RT and no adjuvant RT groups, respectively (Fig. 2). In multivariate analysis, advanced pathological N stage (HR, 2.74; *p* = 0.021), contralateral LN metastasis at diagnosis (HR, 3.24; *p* = 0.019), abutting or positive surgical margins (HR, 2.36; *p* = 0.019), and no adjuvant RT (HR, 0.18; *p* < 0.001) were independent risk factors for LRF (Table 3). Only one of six low-risk patients (stage T1 or 2, > 3-mm surgical margins, pathological stage N0 or 1, and no extranodal extension) who underwent surgery and did not receive induction chemotherapy or adjuvant RT experienced LRF.

**Table 3:** Prognostic factors for locoregional failure in patients treated with primary surgery

	Univariate analysis		Multivariate analysis	
	HR (95% CI)	*p* value	HR (95% CI)	*p* value
Age (< 58 yrs vs. ≥ 58 yrs)	1.04 (0.54–1.99)	0.915		
Tobacco use (≤ 10 PY vs. > 10 PY)	0.68 (0.34–1.34)	0.266		
T stage (T1-2 vs. T3-4)	2.23 (1.07–4.62)	0.031		
Pathological N stage (N0-1 vs. N2)	2.26 (1.14–4.46)	0.019	2.74 (1.17–6.41)	0.021
Contralateral LN metastasis (No vs. Yes)	2.84 (1.25–6.50)	0.013	3.24 (1.21–8.65)	0.019
Extranodal extension (No vs. Yes)	1.61 (0.82–3.14)	0.165		
Lymphovascular invasion (No vs. Yes)	1.61 (0.82–3.16)	0.169		
Perineural invasion (No vs. Yes)	1.06 (0.32–3.45)	0.929		
Surgical margin status (Negative vs. Abutting + Positive)	1.98 (1.02–3.85)	0.043	2.36 (1.15–4.86)	0.019
Adjuvant radiotherapy (No vs. Yes)	0.38 (0.19–0.73)	0.004	0.18 (0.08–0.40)	< 0.001
Concurrent chemoradiotherapy (No vs. Yes)	1.77 (0.52–6.04)	0.362		
Induction chemotherapy (No vs. Yes)	1.34 (0.68–2.64)	0.392		

The foreparts of the parentheses were set as the reference groups in the multivariable analysis

*HR* hazard ratio, *CI* confidence interval, *PY* pack-years, *LN* lymph node

Among the 84 patients who underwent primary surgery without adjuvant RT, 16 (19.0%) experienced LRF during the follow-up period. In the multivariate analysis, advanced pathological T stage (HR, 2.30; *p* = 0.026) was identified as an independent risk factor for LRF in patients who underwent primary surgery without adjuvant RT (Additional file 1: Table S2).

**Fig. 3:**
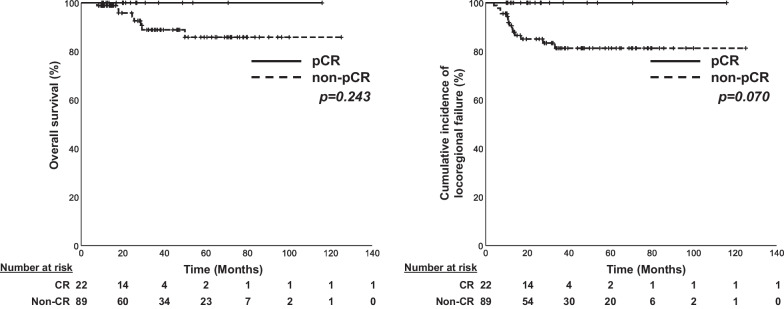
Kaplan–Meier curves for overall survival and locoregional failure stratified by pathologic complete remission versus non-pathologic complete remission. pCR, pathologic complete remission

Of the 111 patients who received induction chemotherapy before primary surgery, pathological complete remission (CR), partial response, stable disease, and progressive disease were found in 22, 66, 18, and 5 patients, respectively. None of the patients with pathological CR died or experienced LRF during the follow-up period (Fig. 3). Further, 15 patients (68.2%) had never received adjuvant RT. Among the 111 patients, LRF occurred in 14, pathological partial response in 11, stable disease in 2, and progressive disease in 1. On multivariate analysis, advanced pathological N stage (HR, 4.17; *p* = 0.051) and no adjuvant RT (HR, 0.27; *p* = 0.062) showed nonsignificant associations with LRF (Additional file 1: Table S3).

## Discussion

Survival was better in patients who underwent primary surgery than in those who underwent primary RT. However, because the primary surgery group tended to have a less advanced stage than the RT group, the results were similar when adjusted for clinical factors. Additionally, in patients who underwent primary surgery, LRF was significantly lower in those who subsequently underwent adjuvant RT than in those who did not. Risk factors for LRF after surgery included advanced pathological N stage, contralateral LN metastasis at diagnosis, and abutting or positive surgical margins. Since advanced T stage is a risk factor for LRF in patients who have not received adjuvant RT after primary surgery, primary RT is a better alternative than surgery for patients with advanced clinical T stage.

The relationship between the response after induction chemotherapy and LRF was not confirmed. Even in patients with a good response, those with advanced N stage should be considered for adjuvant RT. However, in the case of CR after induction chemotherapy, deintensification for primary RT or surgery was considered because none of the patients experienced a relapse.

Surgery and RT can be considered primary treatments for HPV-associated oropharyngeal cancer. The outcomes of both modalities are similar, with overall response rates of over 80% [14]. Since standard surgery for oropharyngeal cancer has historically consisted of major operations with relatively high toxicities, such as lip-split mandibulectomy and the drop-down technique, primary RT was preferred over surgery until the early 2000s [15]. However, since the development of TORS, a minimally invasive surgical technique, surgery boasts similar outcomes to RT, with lesser morbidity and better functional outcomes than those of conventional surgery [16]. A randomized trial (ORATOR) compared primary RT and primary TORS in patients with early-stage HPV-associated oropharyngeal cancer and found no difference in outcomes [8]. However, RT is advantageous because it preserves the head and neck tissues and organs and allows patients to maintain their quality of life and daily activities, such as swallowing and talking.

In patients with HPV-associated oropharyngeal cancer in the early T and N stages, locoregional control and survival exceeded 90%, with no adverse features (negative surgical margins and no lymphovascular invasion, extranodal extension, or perineural invasion), even among those who did not undergo adjuvant RT following surgery [17–20]. According to the results of a randomized phase II trial (E3311), the 2-year PFS rate after TORS was 96.9%, with no observed LRF in low-risk patients (stage T1 or 2, > 3-mm surgical margins, N0 or 1, and no extranodal extension) [21]. In our study, one of six low-risk patients who only underwent surgery experienced LRF. Adjuvant RT may be beneficial in patients with advanced pathologic N stage, contralateral LN metastasis at diagnosis, or abutting or positive surgical margins.

As a deintensification strategy for HPV-associated oropharyngeal cancer treatment, the radiation dose may be reduced or avoided in good responders to induction chemotherapy. Several studies have evaluated dose reduction strategies for primary RT after induction chemotherapy [22, 23]; however, little evidence exists on deintensification strategies for adjuvant RT in patients who underwent surgery after induction chemotherapy. In a recent study on less-invasive surgery after induction chemotherapy, 15.0% of patients received adjuvant RT, and only 4.9% experienced recurrence [24], suggesting that adjuvant RT can be omitted when no adverse histological features are observed. In our institution, induction chemotherapy was used, followed by minimally invasive surgery such as TORS, which maintained the patient's health and function.

In our institution, induction chemotherapy was administered to patients with oropharyngeal cancer based on the results of our clinical trial [25]. Induction chemotherapy maintains the patient's health and function by reducing the surgical extent and avoiding the need for adjuvant treatment depending on the response. In our study, induction chemotherapy had a high response rate of 79.3%. In particular, the outcomes of patients with pathological CR after induction chemotherapy were excellent, and 68.2% of these patients did not receive adjuvant RT. However, even if the response to induction chemotherapy is good, adjuvant RT may be necessary in patients with advanced pathological N stage. To support RT deintensification strategies, the role of induction chemotherapy must be established through larger prospective studies.

The most recent study on a large cohort with HPV-associated tonsil cancer analyzed 1758 patients in the National Cancer Database in 2020 [26]; no significant difference in 3-year survival rates was observed among patients who underwent tonsillectomy with neck dissection, tonsillectomy with neck dissection plus adjuvant RT, tonsillectomy with neck dissection plus adjuvant CCRT, RT alone, or CCRT. Although our study did not analyze early-stage cancer, when the clinical factors between the primary surgery and RT groups were adjusted through PSM, the results were similar.

Many studies have indicated age as a prognostic factor in oropharyngeal cancer, but it was not a significant factor in our study. The main cause of poor prognosis in older patients is failure to complete treatment. However, in our study, patients who did not complete RT were excluded to accurately analyze the results according to the strategy. Therefore, there was no difference in prognosis according to age in our study.

Due to its retrospective nature, this study has several limitations. First, while radiation dose is significant enough to be presented as a deintensification strategy and major factor influencing locoregional control, it was not analyzed because RT strategies varied according to physician preference. Second, as more evidence for deintensification strategies is presented, more patients are treated with surgery alone or a reduced radiation volume. The relatively low failure rate may be due to their short follow-up period. Third, because we analyzed all patients with HPV-associated tonsil cancer treated at our institution, the tumor and treatment characteristics are heterogeneous. Fourth, the surgical extent or technique that may have affected prognosis could not be fully analyzed. Fifth, quality surveys, such as the European Organization for Research Treatment of Cancer (EORTC) QLQ H&N 35 or EORTC QLQ C30, which provide quality of life assessment after treatment, could not be implemented due to the retrospective nature of the study [27, 28]. Sixth, since this was a retrospective study, there is a possibility that the records were not accurate. In particular, in the case of smoking history, detailed smoking history records may be relatively inaccurate because patients who underwent surgery were asked for their smoking history in detail before hospitalization, but patients who underwent RT were not hospitalized. Other important prognostic factors such as performance status and comorbidities were also not included in the analysis due to inaccuracies in the records. Seventh, due to the lack of medical record after RT, patients who did not complete RT were excluded from the analysis. Therefore, it was difficult to analysis on intension to treat basis, and a further prospective study is needed for a more accurate intension to treat analysis. Finally, approximately one-third of all patients received induction chemotherapy, which is not a commonly used treatment strategy in HPV-associated oropharyngeal cancer and may be difficult to apply in other populations.

Despite these limitations, this study is relevant because the examination, treatment, and clinical follow-up routines were consistent as only patients diagnosed and treated at our institution were enrolled and analyzed. In addition, to complement the heterogeneous tumor characteristics, the treatment groups were analyzed after PSM, and a subgroup analysis was performed separately for patients who underwent surgery. Moreover, our study analyzed oropharyngeal cancer subsites in the largest cohort from a single institution among studies on outcomes of patients with HPV-associated tonsil cancer. Finally, the results of induction chemotherapy at our institution indicated the possibility of reducing surgical extent and excluding additional adjuvant treatment.

## Conclusions

Our findings demonstrate that outcomes for primary surgery and primary RT in HPV-positive tonsil cancer were similar after adjusting for clinical factors. Adjuvant RT after surgery significantly reduces LRF, and its effect is greater in cases with advanced pathological N stage, contralateral LN metastasis at diagnosis, and abutting or positive surgical margins. Primary RT might be considered instead of surgery in patients with advanced pathological T stage, a risk factor for LRF in patients who have not received adjuvant RT after primary surgery. After induction chemotherapy, deintensification for adjuvant RT is possible if pathological CR is achieved.

## Supplementary Information

**Additional file 1: Table S1.** Characteristics for patients treated with and without surgery before and after propensity score-matching; **Table S2.** Prognostic factors for locoregional failure in patients treated with primary surgery alone without adjuvant radiotherapy; **Table S3.** Prognostic factors for locoregional failure in patients treated with induction chemotherapy followed by surgery.

## Data Availability

Research data are stored in an institutional repository and are available from the corresponding author upon reasonable request.

## Abbreviations

CCRT: Concurrent chemoradiotherapy
CR: Complete remission
CT: Computed tomography
CTV: Clinical target volume
EQD2: Equivalent dose at 2 Gy per fraction
HPV: Human papillomavirus
HR: Hazard ratio
IQR: Interquartile range
LN: Lymph node
LRF: Locoregional failure
OS: Overall survival
PFS: Progression-free survival
PSM: Propensity score matching
RT: Radiotherapy
TORS: Transoral robotic surgery
