# Supplementary material for: Treatment outcomes and radiotherapy deintensification strategies in human papillomavirus-associated tonsil cancer
Source: Radiat Oncol. 2022 Dec 20;17:209. doi: 10.1186/s13014-022-02177-1 (PMC9764715; doi:10.1186/s13014-022-02177-1)
Supplement: Supplementary file 1 — Additional file 1: Table S1. Characteristics for patients treated with and without surgery before and after propensity score-matching; Table S2. Prognostic factors for locoregional failure in patients treated with primary surgery alone without adjuvant radiotherapy; Table S3. Prognostic factors for locoregional failure in patients treated with induction chemotherapy followed by surgery. [file 13014_2022_2177_MOESM1_ESM.docx]

**Supplementary Table 1.** Characteristics for patients treated with and without surgery before and after propensity score-matching

| Characteristic | Before PSM | | | | | |  | After PSM | | | | | |
| --- | --- | --- | --- | --- | --- | --- | --- | --- | --- | --- | --- | --- | --- |
|  | No surgery (N=66) | | Surgery (N=308) | | p-value | SMD |  | No surgery (N=57) | | Surgery (N=57) | | p-value | SMD |
|  | N | % | N | % |  |  |  | N | % | N | % |  |  |
| Age (years, median [IQR]) | 59 (52-67) | | 58 (52-63) | | 0.250 | 0.160 |  | 59 (51-68) | | 58 (53-63) | | 0.458 | 0.152 |
| Sex |  |  |  |  | 0.693 | 0.053 |  |  |  |  |  | 1.000 | 0.000 |
| Male | 58 | 87.9 | 265 | 86.0 |  |  |  | 49 | 86.0 | 49 | 86.0 |  |  |
| Female | 8 | 12.1 | 43 | 14.0 |  |  |  | 8 | 14.0 | 8 | 14.0 |  |  |
| Tobacco use |  |  |  |  | 0.058 | 0.256 |  |  |  |  |  | 1.000 | 0.000 |
| ≤10 pack-years | 44 | 66.7 | 166 | 53.9 |  |  |  | 39 | 68.4 | 39 | 68.4 |  |  |
| >10 pack-years | 22 | 33.3 | 142 | 46.1 |  |  |  | 18 | 31.6 | 18 | 31.6 |  |  |
| T stage |  |  |  |  | <0.001 | 0.654 |  |  |  |  |  | 1.000 | 0.000 |
| T1 | 12 | 18.2 | 75 | 24.4 |  |  |  | 12 | 21.1 | 12 | 21.1 |  |  |
| T2 | 26 | 39.4 | 186 | 60.4 |  |  |  | 26 | 45.6 | 26 | 45.6 |  |  |
| T3 | 14 | 21.2 | 30 | 9.7 |  |  |  | 11 | 19.3 | 11 | 19.3 |  |  |
| T4 | 14 | 21.2 | 17 | 5.5 |  |  |  | 8 | 14.0 | 8 | 14.0 |  |  |
| Clinical N stage |  |  |  |  | <0.001 | 0.722 |  |  |  |  |  | 1.000 | 0.000 |
| N0 | 4 | 6.1 | 40 | 13.0 |  |  |  | 4 | 7.0 | 4 | 7.0 |  |  |
| N1 | 41 | 62.1 | 242 | 78.6 |  |  |  | 38 | 66.7 | 38 | 66.7 |  |  |
| N2 | 19 | 28.8 | 26 | 8.4 |  |  |  | 15 | 26.3 | 15 | 26.3 |  |  |
| N3 | 2 | 3.0 | 0 | 0.0 |  |  |  | 0 | 0.0 | 0 | 0.0 |  |  |

***Abbreviations:*** PSM, propensity score-matching; SMD, standardized mean difference; IQR, inter-quartile range

**Supplementary Table 2.** Prognostic factors for locoregional failure in patients treated with primary surgery alone without adjuvant radiotherapy

|  | Univariate analysis | | Multivariate analysis | |
| --- | --- | --- | --- | --- |
|  | HR (95% CI) | p-value | HR (95% CI) | p-value |
| Age (<58 yrs vs. ≥58 yrs) | 0.99 (0.56-1.77) | 0.984 |  |  |
| Tobacco use (≤10 pack-years vs. >10 pack-years) | 0.69 (0.38-1.27) | 0.238 |  |  |
| T stage (T1-2 vs. T3-4) | 2.61 (1.43-4.75) | 0.002 | 2.30 (1.10-4.79) | 0.026 |
| Pathological N stage (N0-1 vs. N2) | 2.92 (0.71-12.03) | 0.139 | 4.88 (0.67-35.65) | 0.118 |
| Contralateral LN metastasis (No vs. Yes) | 2.91 (1.50-5.61) | 0.002 |  |  |
| Extranodal extension (No vs. Yes) | 1.60 (0.82-3.13) | 0.168 |  |  |
| Lymphovascular invasion (No vs. Yes) | 1.60 (0.81-3.14) | 0.175 |  |  |
| Perineural invasion (No vs. Yes) | 1.07 (0.33-3.48) | 0.915 |  |  |
| Surgical margin status (Negative vs. Abutting+Positive) | 1.97 (1.02-3.83) | 0.044 |  |  |
| Induction chemotherapy (No vs. Yes) | 1.40 (0.77-2.56) | 0.270 |  |  |

The foreparts of the parentheses were set as the reference groups in the multivariable analysis

***Abbreviations:*** HR, hazard ratio; CI, confidence interval

**Supplementary Table 3.** Prognostic factors for locoregional failure in patients treated with induction chemotherapy followed by surgery

|  | Univariate analysis | | Multivariate analysis | |
| --- | --- | --- | --- | --- |
|  | HR (95% CI) | p-value | HR (95% CI) | p-value |
| Age (<58 yrs vs. ≥58 yrs) | 1.34 (0.46-3.86) | 0.589 |  |  |
| Tobacco use (≤10 pack-years vs. >10 pack-years) | 1.18 (0.41-3.39) | 0.766 |  |  |
| T stage (T1-2 vs. T3-4) | 1.25 (0.35-4.48) | 0.733 |  |  |
| Pathological N stage (N0-1 vs. N2) | 2.13 (0.71-6.37) | 0.175 | 4.17 (1.00-17.48) | 0.051 |
| Contralateral LN metastasis (No vs. Yes) | 1.98 (0.55-7.12) | 0.294 |  |  |
| Extranodal extension (No vs. Yes) | 1.73 (0.60-4.99) | 0.311 |  |  |
| Lymphovascular invasion (No vs. Yes) | 1.77 (0.55-5.64) | 0.336 |  |  |
| Perineural invasion (No vs. Yes) | 1.23 (0.16-9.44) | 0.842 |  |  |
| Surgical margin status (Negative vs. Abutting+Positive) | 1.42 (0.44-4.53) | 0.556 |  |  |
| Adjuvant radiotherapy (No vs. Yes) | 0.51 (0.17-1.48) | 0.212 | 0.27 (0.07-1.07) | 0.062 |
| Concurrent chemoradiotherapy (No vs. Yes) | 2.74 (0.34-22.26) | 0.347 |  |  |
| Pathological response (SD+PD vs. PR+CR) | 0.89 (0.25-3.20) | 0.862 |  |  |

The foreparts of the parentheses were set as the reference groups in the multivariable analysis

***Abbreviations:*** HR, hazard ratio; CI, confidence interval; SD, stable disease; PD, progressive disease; CR, complete remission; PR, partial response
